# Accelerometer-based physical activity is associated with the gut microbiota in 8416 individuals in SCAPIS

**DOI:** 10.1016/j.ebiom.2024.104989

**Published:** 2024-01-31

**Authors:** Gabriel Baldanzi, Sergi Sayols-Baixeras, Elin Ekblom-Bak, Örjan Ekblom, Koen F. Dekkers, Ulf Hammar, Diem Nguyen, Shafqat Ahmad, Ulrika Ericson, Daniel Arvidsson, Mats Börjesson, Peter J. Johanson, J. Gustav Smith, Göran Bergström, Lars Lind, Gunnar Engström, Johan Ärnlöv, Beatrice Kennedy, Marju Orho-Melander, Tove Fall

**Affiliations:** a Molecular Epidemiology, Department of Medical Sciences, Uppsala University, Uppsala, Sweden; b CIBER Cardiovascular Diseases (CIBERCV), Instituto de Salud Carlos III, Madrid, Spain; c Department of Physical Activity and Health, The Swedish School of Sport and Health Sciences, Stockholm, Sweden; d Preventive Medicine Division, Harvard Medical School, Brigham and Women’s Hospital, Boston, MA, United States; e Department of Clinical Sciences in Malmö, Lund University, Malmö, Sweden; f Center for Health and Performance, Department of Food and Nutrition, and Sport Science, University of Gothenburg, Gothenburg, Sweden; g Center for Lifestyle Intervention, Department of Molecular and Clinical Medicine, University of Gothenburg, Gothenburg, Sweden; h Department of Medicine, Geriatric and Acute Medicine Östra, Sahlgrenska University Hospital, Gothenburg, Sweden; i Occupational and Environmental Medicine, Department of Medical Sciences, Uppsala University, Uppsala, Sweden; j Occupational and Environmental Medicine, Uppsala University Hospital, Uppsala, Sweden; k The Wallenberg Laboratory/Department of Molecular and Clinical Medicine, Institute of Medicine, Gothenburg University and the Department of Cardiology, Sahlgrenska University Hospital, Gothenburg, Sweden; l Department of Cardiology, Clinical Sciences, Lund University and Skåne University Hospital, Lund, Sweden; m Wallenberg Center for Molecular Medicine and Lund University Diabetes Center, Lund University, Lund, Sweden; n Department of Molecular and Clinical Medicine, Institute of Medicine, Sahlgrenska Academy, University of Gothenburg, Gothenburg, Sweden; o Department of Clinical Physiology, Sahlgrenska University Hospital, Region Västra Götaland, Gothenburg, Sweden; p Clinical Epidemiology, Department of Medical Sciences, Uppsala University, Uppsala, Sweden; q Division of Family Medicine and Primary Care, Department of Neurobiology, Care Science and Society, Karolinska Institutet, Huddinge, Sweden; r School of Health and Social Studies, Dalarna University, Falun, Sweden

**Keywords:** Accelerometery, Gastrointestinal microbiome, Exercise, Sedentary behaviour, Epidemiology

## Abstract

**Background:**

Previous population-based studies investigating the relationship between physical activity and the gut microbiota have relied on self-reported activity, prone to reporting bias. Here, we investigated the associations of accelerometer-based sedentary (SED), moderate-intensity (MPA), and vigorous-intensity (VPA) physical activity with the gut microbiota using cross-sectional data from the Swedish CArdioPulmonary bioImage Study.

**Methods:**

In 8416 participants aged 50–65, time in SED, MPA, and VPA were estimated with hip-worn accelerometer. Gut microbiota was profiled using shotgun metagenomics of faecal samples. We applied multivariable regression models, adjusting for sociodemographic, lifestyle, and technical covariates, and accounted for multiple testing.

**Findings:**

Overall, associations between time in SED and microbiota species abundance were in opposite direction to those for MPA or VPA. For example, MPA was associated with lower, while SED with higher abundance of *Escherichia coli*. MPA and VPA were associated with higher abundance of the butyrate-producers *Faecalibacterium prausnitzii* and *Roseburia* spp. We observed discrepancies between specific VPA and MPA associations, such as a positive association between MPA and *Prevotella copri*, while no association was detected for VPA. Additionally, SED, MPA and VPA were associated with the functional potential of the microbiome. For instance, MPA was associated with higher capacity for acetate synthesis and SED with lower carbohydrate degradation capacity.

**Interpretation:**

Our findings suggest that sedentary and physical activity are associated with a similar set of gut microbiota species but in opposite directions. Furthermore, the intensity of physical activity may have specific effects on certain gut microbiota species.

**Funding:**

10.13039/501100000781European Research Council, 10.13039/501100003793Swedish Heart-Lung Foundation, 10.13039/501100004359Swedish Research Council, 10.13039/501100004063Knut and Alice Wallenberg Foundation.

**Research in context**

**Evidence before this study**

We searched PubMed for publications since 2014 on “physical activity and gut microbiota”; “physical activity and microbiome”; “sedentary behaviour and gut microbiota”; and “sedentary behaviour and microbiome”. We also checked reference lists in the identified articles for additional studies. Overall, intervention studies and population-based studies reported a positive association between the practice of physical activity and the abundance of butyrate producers in the gut. However, other associations were largely inconsistent. Only one previous large population-based study was identified. This study used predominantly self-reported physical activity data and the gut microbiota was assessed using 16S ribosomal RNA gene, which has a low taxonomic resolution.

**Added value of this study**

This study stands out as the largest population-based investigation thus far exploring the association of accelerometer-based sedentary behaviour and physical activity with the gut microbiota composition and functional profile. Accelerometers are considered to provide a more valid estimate of physical activity than self-reported data. Moreover, the gut microbiota was assessed with shotgun metagenomics, which has marked advantages in determining the abundance of individual species and profiling the functional capacity of the microbiota. Our findings indicate that sedentary behaviour and physical activity are linked to the abundance of more than 600 species. In certain cases, moderate-intensity and vigorous-intensity activity showed dissimilar associations with the same species. Additionally, sedentary behaviour was associated with a microbiota with lower capacity to degrade carbohydrates, particularly from dietary fibres.

**Implications of all the available evidence**

Our study shows that the time spent in sedentary behaviour or physical activity of different intensities is connected to the abundance of a large part of the gut microbiota species and the overall functional profile. These findings suggest that physical activity could modify the gut microbiota composition with potential repercussions for host health. Further longitudinal and experimental studies are needed to explore these relationships.

## Introduction

Physical activity has well-established health benefits, including reduced risk for cardiovascular disease, type 2 diabetes,^1^^,^^2^ and psychiatric conditions like depression.^3^ Conversely, sedentary behaviour (SED), defined as sitting or non-sleep lying activities with low energy expenditure, is associated with an increased risk of type 2 diabetes and cardiovascular mortality.^4^, ^5^, ^6^ Some studies have though indicated that the risks attributed to SED could be substantially attenuated by physical activity at high intensities.^7^, ^8^, ^9^ The mechanisms linking physical activity to health benefits are various. Recently, it has been suggested that certain benefits of physical activity might be mediated by changes on the gut microbiota.^10^

The gut microbiota is a community of microorganisms within the gastrointestinal tract, that interacts with the host.^11^ Evidence suggests that the gut microbiota plays a role in the development of type 2 diabetes, cardiovascular diseases, and psychiatric conditions.^12^, ^13^, ^14^, ^15^ Gut microbiota may influence brain homeostasis through the microbiota-gut-brain axis, which includes microbe-produced neurotransmitters and other molecules that can affect the central nervous system via neuronal pathways or the immune system.^16^

Regular physical activity may affect the gut microbiota through various mechanisms, including modulation of the gut immune system, reduction in the intestinal transit time^17^ and splanchnic blood flow, transient increase in the intestinal permeability,^18^ and modulation of the enterohepatic circulation of bile acids.^19^ Smaller intervention studies in specific populations have reported changes to the gut microbiota composition after structured exercise, with a decrease in *Clostridium* and *Blautia* and an increase in *Bifidobacterium* and *Dorea*.^20^, ^21^, ^22^ In population-based studies, self-reported moderate (MPA) and vigorous-intensity (VPA) physical activity have been associated with increased gut microbiota diversity,^23^ and SED with increased abundance of *Roseburia hominis* and *Erysipelatoclostridium* species.^24^ However, these studies had limited microbiota taxonomic resolution and used self-reported physical activity information, which may be affected by reporting bias.^25^ Therefore, there is a need for larger population-based studies that combine sensor-based physical activity assessment with gut microbiota profiling in high taxonomic resolution. Here, we aimed to identify associations of accelerometer-based SED and physical activity with the gut microbiota analysed with deep shotgun metagenomics, in 8416 participants, using cross-sectional data from the Swedish CArdioPulmonary BioImage Study (SCAPIS).

## Methods

### Study population

The SCAPIS cohort includes 30 154 women and men aged 50–65 enrolled between 2013 and 2018 to the baseline investigation, which was conducted at six sites in Sweden.^26^ A random selection of the target population was invited to the study and 50.3% agreed to participate. Participants answered a comprehensive questionnaire on lifestyle, diet and health history, and underwent an extensive set of clinical examinations, including computed tomography of coronary artery, lungs, and the abdomen. Coronary atherosclerosis was detected in 42.1% of the participants without known coronary artery disease.^26^ The baseline investigation also included the wear of a hip accelerometer for seven days. Participants from Malmö and Uppsala were also invited to provide faecal samples for metagenomic analysis (n = 9831).^27^

### Accelerometer data processing

Detailed information on the accelerometer data processing in SCAPIS has been published by Ekblom-Bak et al.^28^ In this study, we applied the same protocol with the refinement of excluding registrations during estimated time in bed. Participants were instructed to wear the ActiGraph tri-axial accelerometer (GT3X+, wGT3X+, or wGT3X-BT, Actigraph LCC, Pensacola, USA) over the hip for seven consecutive days during the whole day, except during sleep and water-based activities. Despite the instructions to remove the hip-worn accelerometer during sleep, 1480 participants (1319 from Uppsala) had unreasonably high average daily accelerometer wear time; i.e., >18 h/day. This indicates that many participants did not remove the accelerometer during sleep. This might be due to other instruments that Uppsala participants were instructed to keep during sleep, such as a thigh-worn acceleromete, a 24-h blood pressure monitor, and a respiratory polygraph. Therefore, we used information from the thigh-worn accelerometer, which was used concomitantly with the hip-worn accelerometer by Uppsala participants, to filter the hip-worn accelerometer data. The thigh-worn accelerometer data from 3780 Uppsala participants was processed with the custom-made software ActiPASS version 1.42 (www.github.com/Ergo-Tools/ActiPASS). ActiPASS uses the rotation and angle of the thigh in relation to the gravity line to identify the time in prolonged lying bouts.^29^ This was used as a proxy for time in bed. Based on this information, we filtered the hip-worn accelerometer data of all Malmö and Uppsala participants to registrations between 5:52 a.m. and 11:46 p.m. in weekdays and 7:15 a.m. and 11:59 p.m. in weekends, which corresponded to the first quartile of end of time in bed and the third quartile of start of time in bed based on thigh-accelerometer data.

Raw accelerometer data were transformed into counts per minute (cpm) over 60s epochs. SED, low-intensity physical activity (LIPA), MPA, and VPA were defined using previously validated cut-offs^30^ as <200 cpm, 200–2689 cpm, 2690–6166 cpm, and ≥6167 cpm, respectively. Non-wear time was defined as periods of ≥60 min with zero counts, with intervals of maximum 2 min of 0–199 cpm. A valid day was defined as a day with >10 h of wear time. We excluded 397 participants who had <4 valid days. The percentage of time in SED, LIPA, MPA, or VPA were calculated by dividing the time spent in each activity by the total wear time.^28^ Given the compositional structure of time spent in SED, LIPA, MPA, or VPA, we applied an additive log-ratio (alr) transformation, a compositional data analysis method.^31^ In this transformation, LIPA was chosen as the reference component, as this choice produced the least correlated coordinates (Supplementary Fig. S1). Moreover, in mutually adjusted models for alr-transformed variables, the estimates for MPA and VPA are the same, regardless of whether LIPA or SED is used as the reference component. To address the zeros in VPA, we have added 0.1 to VPA before the alr-transformation. To correct for data distortions resulting from the replacement of zeros,^32^ after the alr-transformation, the initial zero values were replaced with the minimal non-zero value transformed. The alr-transformed SED, MPA, and VPA were used for the subsequent analysis.

### Faecal metagenomics

The gut microbiota was assessed through metagenomic analyses of faecal samples using a previously described protocol.^27^ In summary, faecal samples were collected at home, kept in the home freezer until the second study visit, and stored at −80 °C until shipped to Clinical Microbiomics A/S (Copenhagen, Denmark) for DNA extraction, library preparation, sequencing with Ilumina Novaseq 6000 (Illumina, CA, USA), bioinformatics processing, and taxonomic annotation. Metagenomic species were defined by binning of co-abundance genes, as previously described.^33^ Alpha diversity, a metric of the diversity of species within a sample, was estimated using the Shannon diversity index, inverse Simpson index, and richness (number of species). Beta diversity, a metric of the composition dissimilarity between samples, was estimated using the Bray–Curtis dissimilarity.^34^ These estimations were performed with the R package *vegan* on sequence data that was rarefied to 210 430 read-pairs for all samples to account for the differences in sequencing depth. The functional potential of the gut microbiota was defined by the abundance of genes of the manually curated gut metabolic modules (GMM, version 1.07)^35^ and microbiota-gut-brain modules (MGB, version 1.0).^16^ We chose to also include the MGB modules given that both physical activity and the gut microbiota have been associated with mental and neurological health. GMMs capture the microbiota metabolic potential and anaerobic fermentation capacity,^35^ while MGBs comprise the capacity to degrade or produce potentially neuroactive compounds.^16^ To calculate the abundance of respective modules, we used the R package *Omixer-RPM* version 0.3.2^36^ considering a minimum module coverage of 66.6%. Analyses were focused on species with a relative abundance >0.01% in ≥1% of individuals, and GMMs and MGBs present in ≥1% of individuals, resulting in 1335 species, 90 GMMs, and 44 MGBs for further analysis. Species, GMM, and MGB were centered log-ratio transformed. To deal with zero values, 0.001% was added to the relative abundance of species and 0.00001% was added to the GMMs and MGBs. After the transformation, the initial zero values were replaced with the minimal transformed value produced by the non-zero values for the respective species or modules. This replacement ensures that all values that were equal to zero before the transformation will have an equally low value after the transformation. For details, see Supplemental methods.

### Covariates

Covariate information was obtained from the SCAPIS questionnaire, and anthropometric measurements and fasting plasma samples collected during study site visit. Mean daily intake of alcohol, fruit and vegetables, whole grain, protein, and total energy were estimated from the food frequency questionnaire.^37^^,^^38^ Protein intake was transformed to percentages of non-alcohol energy intake. We categorized smoking status as current, former, or non-smoker, and highest achieved education level as incomplete compulsory, complete compulsory, secondary, or university education. Country of birth was grouped as Scandinavia (Sweden, Denmark, Norway, or Finland), non-Scandinavian Europe, Asia, and other countries. Information on medications prescribed for hypertension, type 2 diabetes, dyslipidaemia, depression, and anxiety within six months before the first site visit was retrieved from the Swedish Prescribed Drug Register (Supplemental Methods). Proton pump inhibitor usage was defined as a measurable level of omeprazole or pantoprazole metabolites in plasma. Antibiotic use was defined as a dispensed prescription (Anatomical Therapeutic Chemical code J01) up to three months before the first visit.

### Statistical method

#### Model specification

Based on current literature,^23^^,^^28^^,^^39^ we created a directed acyclic graph (DAG) prior to the analysis phase using the DAGitty tool (www.dagitty.net, Supplementary Fig. S2), and selected potential confounders for the main model based on the d-separation criteria.^40^ We added an unobserved variable of “health consciousness” to contribute to our rationale while constructing the DAG. The main model comprised age, sex, alcohol intake, smoking, education, country of birth, study site, month of accelerometer wear, whole grain, fruit and vegetable, protein, and total energy intake. We also adjusted for technical variation covariates including total wear time, percentage of wear time on weekend, and faecal DNA extraction plate. To identify associations independent of adiposity, we explored an additional model further adjusted for body mass index (BMI) and waist-hip ratio (WHR), named adiposity model. Participants with missing information on model covariates were excluded from the respective analysis (complete case analysis). For all analyses, the alr-transformed variables SED, MPA, and VPA were standardized by subtracting the mean and dividing by the standard deviation. Consequently, the linear regression coefficients for these variables will represent expected changes in the outcome by, for example, standard-deviation changes in the log of the ratio of SED to LIPA, while maintaining the ratios of MPA and VPA to LIPA constant. Statistical analyses were conducted in R version 4.1.1 (http://www.r-project.org).

#### Alpha and beta diversity

For alpha diversity, we applied linear regression models with the alpha diversity metrics as the dependent variable and alr-transformed SED, MPA, and VPA jointly as independent variables, while adjusting for covariates of the main or adiposity model. For beta diversity, we used partial distance-based redundancy analysis to estimate the proportion of the interindividual gut microbiota dissimilarity explained by SED, MPA, and VPA after adjustment for covariates. To perform these analyses, we used the function “capscale” (R package *vegan*) with the option “condition”. The p-values were calculated based on 9999 permutations.

#### Species, gut metabolic modules, and microbiota-gut-brain modules

To identify microbiota features associated with the composition of the awake time spent in SED and physical activity, we applied a series of linear regression models with each species, GMM, and MGB introduced as the dependent variable, one at the time, and alr-transformed SED, MPA, and VPA jointly introduced as independent variables together with the main model covariates. We used the likelihood ratio test to compare this model to the alternative model that only contained the covariates. To control for multiple testing, we applied the Benjamini-Hochberg method with a 5% false discovery rate and reported significance as q-values.^41^ To identify associations potentially caused by single influential observations, we calculated dfbetas for SED, MPA, and VPA. If removing the observation with the highest dfbeta resulted in a likelihood ratio test p-value ≥ 0.05, we discarded the association. For the species, GMMs, and MGBs identified with the likelihood ratio test (q-value < 0.05), we examined the individual regression coefficients for SED, MPA, and VPA mutually adjusted in the main model and in the adiposity model. To examine multicollinearity, we have also assessed the variance inflation factor (VIF). To test the heterogeneity of regression coefficients for VPA and MPA for the species associated with either one of these two in the main model, we performed a model-based post-hoc test using the function “linearHypothesis” in the R package *car*. Lastly, the species associated with the composition of awake time-use were examined in two sensitivity analyses: (1) additional adjustment for use of proton-pump inhibitors and medication for hypertension, type 2 diabetes, dyslipidaemia, anxiety, and/or depression; and (2) exclusion of participants that were prescribed antibiotic treatment within the last three months.

### Role of the funding source

The study sponsors had no role in the study design, collection, analysis, or interpretation of data, writing the manuscript, or decision to submit it for publication.

### Ethics

The Swedish Ethical Review Authority approved the SCAPIS study (DNR 2010-228-31M) and the present study (DNR 2018-315 with amendment DNR 2020–06597). All participants provided written informed consent.

## Results

### Study population

**Fig. 1:**
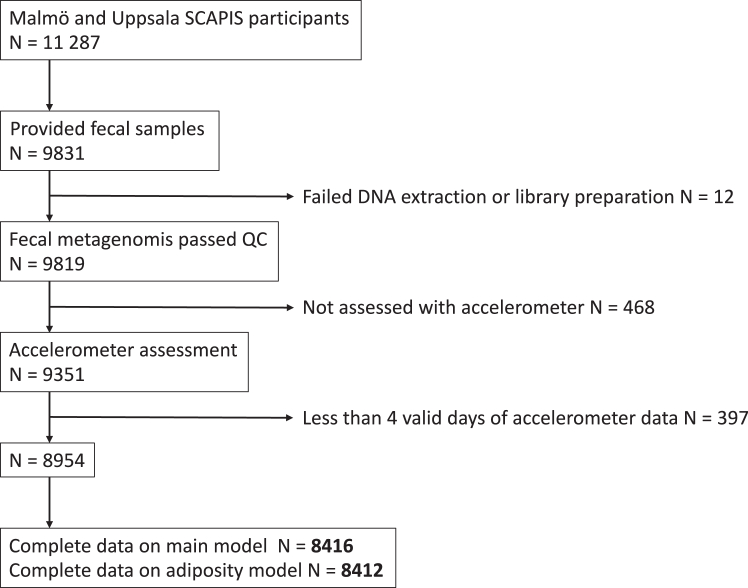
**Flowchart of participants included in this study**.

The study population consisted of 4144 participants from Malmö and 4272 participants from Uppsala with valid accelerometer data, faecal metagenomics data, and complete information on the main model covariates (Table 1). A flowchart with inclusion and exclusion of participants is presented in Fig. 1.

**Table 1:** Characteristics of the study population stratified by study site.

	All	Malmö	Uppsala
	N = 8416	N = 4144	N = 4272
Age, years	57.6 [53.8; 61.4]	57.4 [53.7; 61.2]	57.8 [53.9; 61.5]
Women, N (%)	4485 (53.3%)	2247 (54.2%)	2238 (52.4%)
Accelerometer:			
Wear time in sedentary, %	55.5 [48.5; 62.0]	55.6 [48.7; 62.3]	55.4 [48.4; 61.7]
Wear time in moderate-intensity, %	4.7 [3.2; 6.6]	4.3 [2.8; 6.3]	5.1 [3.6; 6.9]
Wear time in vigorous-intensity, %	0.06 [0.00; 0.48]	0.03 [0.00; 0.37]	0.10 [0.02; 0.61]
Valid days, days	7.0 [6.0; 7.0]	7.0 [6.0; 7.0]	7.0 [7.0; 7.0]
Wear time/day, h	14.5 [13.7; 15.4]	14.1 [13.3; 14.8]	15.0 [14.1; 16.0]
Wear time registered during weekends, %	28.6 [28.6; 28.6]	28.6 [28.6; 28.6]	28.6 [28.6; 28.6]
Alcohol intake, g/day	5.4 [1.8; 10.3]	5.2 [1.6; 10.4]	5.6 [2.0; 10.2]
Smoking, N (%):			
Never	4345 (51.6%)	1814 (43.8%)	2531 (59.2%)
Former	3013 (35.8%)	1633 (39.4%)	1380 (32.3%)
Current	1058 (12.6%)	697 (16.8%)	361 (8.5%)
Education, N (%):			
Compulsory^a^	763 (9.1%)	452 (10.9%)	311 (7.3%)
Upper secondary	3743 (44.5%)	1969 (47.5%)	1774 (41.5%)
University	3910 (46.5%)	1723 (41.6%)	2187 (51.2%)
Country of birth, N (%):			
Scandinavia^b^	7118 (84.6%)	3272 (79.0%)	3846 (90.0%)
Europe	742 (8.8%)	577 (13.9%)	165 (3.9%)
Asia	371 (4.4%)	205 (4.9%)	166 (3.9%)
Other	185 (2.2%)	90 (2.2%)	95 (2.2%)
Dietary variables			
Total energy intake, kcal/day	1594 [1239; 2049]	1572 [1221; 2059]	1611 [1268; 2040]
Protein, E%	16.6 [14.7; 18.6]	16.5 [14.5; 18.7]	16.7 [15.0; 18.6]
Fruit and vegetable, g/day	278.7 [161.2; 436.9]	283.6 [162.8; 453.1]	273.0 [159.5; 421.9]
Whole grain, g/day	31.3 [14.0; 54.5]	28.6 [11.9; 51.4]	34.4 [17.2; 57.3]
BMI and waist-hip ratio	N = 8412	N = 4140	N = 4272
BMI, kg/m^2^	26.4 [24.0; 29.4]	26.6 [24.1; 29.6]	26.3 [24.0; 29.1]
Waist-hip ratio	0.9 [0.9; 1.0]	0.9 [0.8; 1.0]	0.9 [0.9; 1.0]
Sensitivity analysis—medication use	N = 7483	N = 3256	N = 4227
Type 2 diabetes, N (%)	295 (3.9%)	143 (4.4%)	152 (3.6%)
Hypertension, N (%)	1801 (24.1%)	822 (25.2%)	979 (23.2%)
Dyslipidaemia, N (%)	784 (10.5%)	371 (11.4%)	413 (9.8%)
Depression, N (%)	776 (10.4%)	335 (10.3%)	441 (10.4%)
Anxiety, N (%)	269 (3.6%)	139 (4.3%)	130 (3.1%)
Proton pump inhibitors, N (%)	281 (3.8%)	153 (4.7%)	128 (3.0%)
Sensitivity analysis—antibiotic use	N = 8416	N = 4144	N = 4272
Antibiotic last three months, N (%)	498 (5.9%)	270 (6.5%)	228 (5.3%)

Continuous variables described as median and 25^th^ and 75^th^ percentiles. Categorical variables described as absolute numbers and percentages.

a Incomplete or complete compulsory education.

b Sweden, Denmark, Finland or Norway. E%: percentages of non-alcohol energy intake.

### Sedentary behaviour and physical activity associations with alpha diversity and beta diversity

**Fig. 2:**
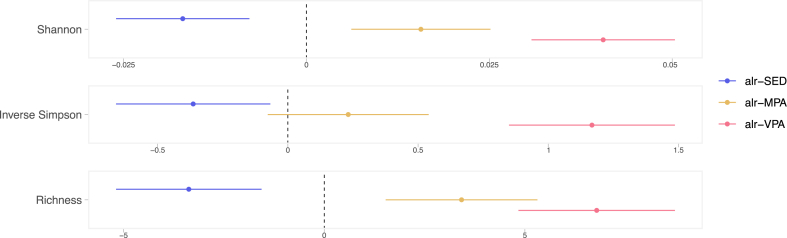
**Association between additive-log ratio (alr) transformed SED, MPA, and VPA with alpha diversity metrics.** Effect estimates show changes in the alpha diversity metrics by standard-deviation changes in the log of the ratios of either SED, MPA, or VPA to LIPA, while keeping the other ratios constant. Associations adjusted for age, sex, alcohol intake, smoking, education, country of birth, study site, month of accelerometer wear, whole grain, fruit and vegetable, protein, and total energy intake, total accelerometer wear time, percentage of wear time on weekend, and faecal DNA extraction plate. Circles and bars represent the regression coefficients and the 95% confidence intervals, respectively. SED: time in sedentary behaviour; MPA: time in moderate-intensity physical activity; VPA: time in vigorous-intensity physical activity.

We found that the proportion of time spent in SED was independently associated with lower alpha diversity in all metrics, whereas time spent in MPA and VPA were independently associated with higher Shannon diversity and richness in the main model (Fig. 2 and Supplementary Table S1). VPA was also associated with higher inverse Simpson index. In the adiposity model, the association between SED and alpha diversity was no longer evident, but MPA and VPA continued to be positively associated with alpha diversity. The highest VIF was 1.36 for VPA in the adiposity model.

To evaluate the contribution of the composition of awake time spent in SED or physical activity on the interindividual variation of the gut microbiota composition, we performed distance-based redundancy analysis on the Bray–Curtis dissimilarly matrix. In the main model, the adjusted R^2^ for alr-transformed SED, MPA and VPA jointly was 0.184% (p-value = 1 × 10^−4^). The individual contribution of SED was 0.054%, MPA 0.043%, and VPA 0.066% (p-value = 1 × 10^−4^, 1 × 10^−4^, and 6 × 10^−4^, respectively). The jointed adjusted R^2^ was 0.160% in the adiposity model (p-value = 1 × 10^−4^). For comparison, the R^2^ for BMI without accounting for other variables was 0.76%, for fruit and vegetables, and whole grain intake 0.49%, and for the dietary variables combined 0.65%.

### Sedentary behaviour and physical activity were associated with a large part of the gut microbiota species

**Fig. 3:**
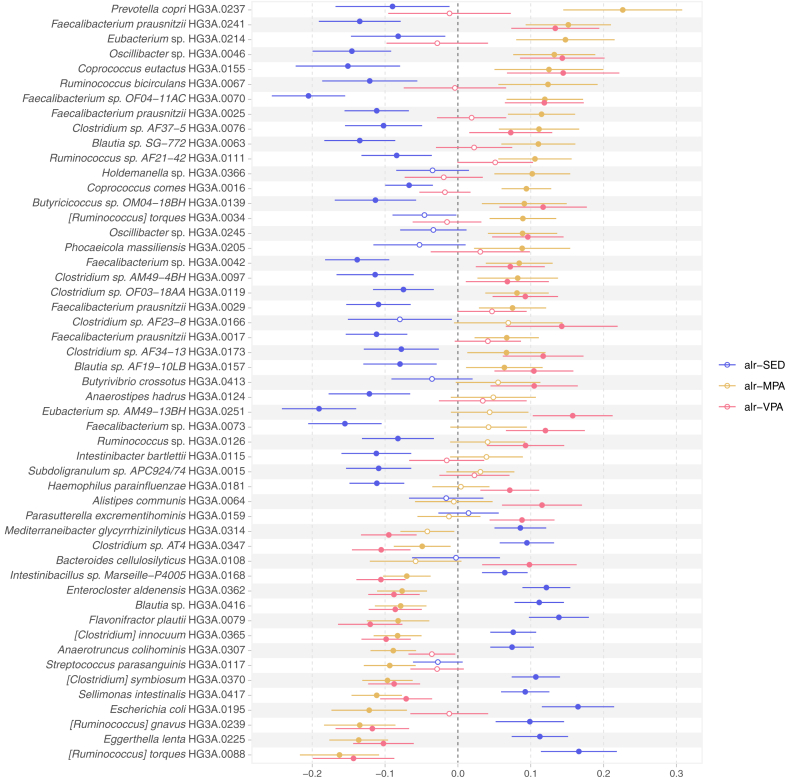
**Species annotated to the genus or specie level representing the top 25 associations for either additive-log ratio (alr) transformed SED, MPA, or VPA based on the absolute regression coefficients.** The effect estimates show changes in the centered-log ratio transformed species abundances by standard-deviation changes in the log of the ratios of either SED, MPA, or VPA to LIPA, while keeping the other ratios constant. Associations adjusted for age, sex, alcohol intake, smoking, education, country of birth, study site, month of accelerometer wear, whole grain, fruit and vegetable, protein, and total energy intake, total accelerometer wear time, percentage of wear time on weekend, and faecal DNA extraction plate. Circles and bars represent the regression coefficients and the 95% confidence intervals, respectively. Filled circles indicate associations with q-values < 0.05. SED: time in sedentary behaviour; MPA: time in moderate-intensity physical activity; VPA: time in vigorous-intensity physical activity.

We identified 651 species associated with the composition of awake time spent in SED and physical activities of different intensities (Supplementary Table S2). None of these associations was attributed to an influential observation. In the main model, the proportion of time spent in SED was independently associated with 309 species (q-value < 0.05), MPA with 322 species, and VPA with 359 species (Supplementary Table S3). Overall, SED, MPA, and VPA were associated with a similar set of species, but regression coefficients for SED were of opposite direction from the MPA and VPA coefficients (Fig. 3). The Spearman correlation was −0.76 between the SED and MPA main model regression coefficients, and −0.72 between the SED and VPA coefficients. Amongst the largest coefficients, MPA was positively associated with *Prevotella copri* and a *Faecalibacterium prausnitzii* subspecies (internal identifier HG3A.0241). Additionally, SED was positively, and MPA and VPA were negatively associated with *Ruminococcus torques, R. gnavus*, and *Eggerthella lenta*. The largest positive coefficients for VPA were with five unclassified species, all belonging to the order *Eubacteriales*. The highest VIF in the main model regressions was 1.33 for VPA (Supplementary Table S3).

Regression coefficients for MPA and VPA were largely consistent in direction. However, for 19 species, there were marked differences in the association with MPA or VPA in the model-based post-hoc test (Supplementary Table S4). For instance, we observed a marked difference for *P. copri* (β MPA = 0.23, β VPA = −0.01, heterogeneity q-value = 0.03). This species had the largest positive coefficient for MPA, but no association was detected with VPA.

The main model and the adiposity model regression coefficients were highly correlated for MPA and VPA (Spearman correlation > 0.95, Supplementary Fig. S3 and Table S2) and slightly less for SED (Spearman correlation = 0.88). The highest VIF in the adiposity model regressions was 1.37 for VPA.

To investigate the potential effect of medication usage on the associations identified in the main model between the composition of awake time in SED or physical activity and 651 species (q-value < 0.05), we performed further adjustment for use of proton pump inhibitors and medications for hypertension, type 2 diabetes, dyslipidaemia, anxiety, and/or depression. After this adjustment, we could detect 565 associated-species (q-value < 0.05). In the sensitivity analysis removing 498 participants who had used any antibiotic in the last three months, 648 species remained associated after adjustment for the main model covariates (q-value < 0.05) (Supplementary Table S5).

### Sedentary behaviour and physical activity are associated with the gut microbiota functional potential

**Fig. 4:**
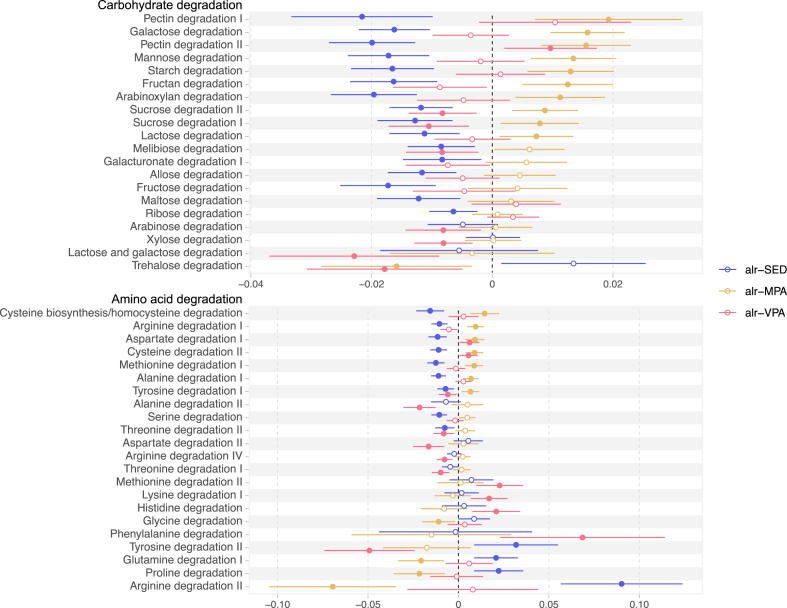
**Association between additive-log ratio (alr) transformed SED, MPA, and VPA with functional modules of carbohydrate or amino acid degradation.** The effect estimates show changes in the centered-log ratio transformed modules abundances by standard-deviation changes in the log of the ratios of either SED, MPA, or VPA to LIPA, while keeping the other ratios constant. Associations adjusted for age, sex, alcohol intake, smoking, education, country of birth, study site, month of accelerometer wear, whole grain, fruit and vegetable, protein, and total energy intake, total accelerometer wear time, percentage of wear time on weekend, and faecal DNA extraction plate. Circles and bars represent the regression coefficients and the 95% confidence intervals, respectively. Filled circles indicate associations with q-values < 0.05. SED: time in sedentary behaviour; MPA: time in moderate-intensity physical activity; VPA: time in vigorous-intensity physical activity.

The functional potential was determined by the abundance of previously curated functional modules, which include modules of carbohydrate, amino acid, or lipid degradation,^35^ and modules of the microbiota-gut-brain axis.^16^ We identified 90 modules associated with the composition of time spent in SED and physical activity. None of the associations were deemed to be due to the effect of an influential observation (Supplementary Table S6). Out of 21 modules of carbohydrate degradation, SED was associated with lower abundance of 16 modules in the main model, while MPA was associated with higher abundance of 10 (Fig. 4 and Supplementary Table S7). With regards to the modules of amino acid degradation, SED was associated with higher and MPA with lower abundance of arginine degradation II, proline degradation, and glutamine degradation I. Among the associations with VPA, we found a positive association with phenylalanine degradation and a negative association with tyrosine degradation.

**Fig. 5:**
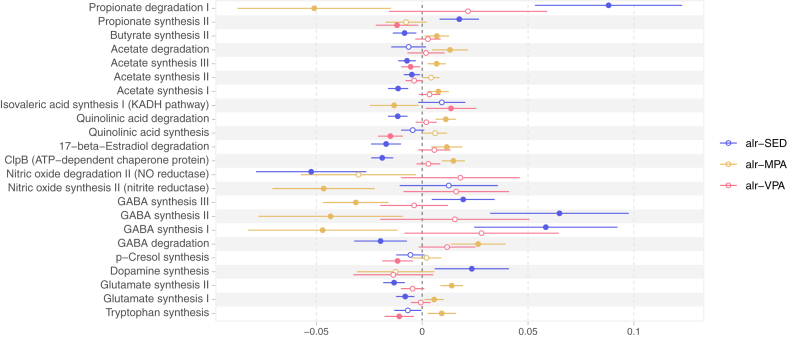
**Association between additive-log ratio (alr) transformed SED, MPA, and VPA with microbiota-gut-brain modules.** The effect estimates show changes in the centered-log ratio transformed modules abundances by standard-deviation changes in the log of the ratios of either SED, MPA, or VPA to LIPA, while keeping the other ratios constant. Associations adjusted for age, sex, alcohol intake, smoking, education, country of birth, study site, month of accelerometer wear, whole grain, fruit and vegetable, protein, and total energy intake, total accelerometer wear time, percentage of wear time on weekend, and faecal DNA extraction plate. Circles represent the regression coefficients, and bars represent the 95% confidence intervals. Filled circles indicate associations with q-values < 0.05. SED: time in sedentary behaviour; MPA: time in moderate-intensity physical activity; VPA: time in vigorous-intensity physical activity.

Among the modules of the microbiota-gut-brain axis, we found that SED was associated with higher and MPA with lower abundance of all three modules of GABA synthesis in the main model (Fig. 5 and Supplementary Table S7). Regarding the modules of short-chain fatty acid (SFCA) metabolism, VPA was associated with lower abundance of propionate synthesis II, while SED was associated with higher abundance of this module and lower abundance of all modules of acetate synthesis. For MPA, we observed associations with higher abundance of modules of acetate synthesis I and III, and butyrate synthesis II. In the adiposity model, we could not detect the association between MPA and butyrate synthesis II or between VPA and propionate synthesis II. The Spearman correlation between the main model and the adiposity model coefficients for SED, MPA, and VPA were 0.86, 0.99, and 0.86, respectively (Supplementary Fig. S3).

## Discussion

In this largest-to-date population-based study of physical activity with gut microbiome encompassing 8416 individuals, we found that, from the 1335 species investigated, 638 (47.8%) were associated with the composition of awake time spent in sedentary behaviour or in physical activities of different intensities. Overall, SED and MPA/VPA were associated with a similar set of species but with regression coefficients in opposite directions. Similar results were observed after adjustment for BMI and WHR (Supplementary Fig. S3). Furthermore, MPA and VPA had concordant associations with gut microbiota species, although with notable exceptions. Additionally, SED, MPA, and VPA were associated with specific microbial functions, some potentially involved in the microbiota-gut-brain axis.

Among the strongest findings with species-level annotations, the proportion of time spent in MPA was associated with higher abundance of *P. copri* and *F. prausnitzii* (HG3A.0241). Time spent in exercise has previously been associated with higher *P. copri* abundance in cyclists.^42^ Nevertheless, the relationship between *P. copri* and health outcomes have been inconsistent. One study suggested a role in fibre fermentation and improved glucose metabolism,^43^ while others reported associations with insulin resistance^44^ and hypertension.^45^ MPA and VPA had similar associations, but with some clear exceptions. MPA was associated with higher abundance of *P. copri* and the *F. prausnitzii* subspecies HG3A.0025, while we could not detect the same associations for VPA. However, a positive association was observed between VPA and the *F. prausnitzii* subspecies HG3A.0241. In total, we identified five *F. prausnitzii* subspecies negatively associated with SED and positively associated with MPA in the main model. In general, *F. prausnitzii* are considered to have anti-inflammatory properties^46^ and individuals with type 2 diabetes have been reported to have a lower abundance of this bacteria.^47^ An increased abundance of *F. prausnitzii* has also been reported in active premenopausal women (n = 40)^48^ and positively associated with time spent in moderate-to-vigorous physical activity (MVPA) in overweight and obese individuals (n = 124),^49^ based on accelerometer assessment. *F. prausnitzii* is an important producer of butyrate, a SCFA that serves as a main energy source for colonocytes and is critical for gut homeostasis.^50^ Moreover, we observed that VPA was associated with higher abundance of *Roseburia* species (*Roseburia* sp. OM04-15AA, *Roseburia* sp. AM16-25, and *Roseburia* sp. AM59-24XD), which are also butyrate producers.

In our analyses, MPA was associated with higher abundance of a butyrate synthesis module, while VPA was associated with higher abundance of propionate synthesis in the main model but not in the adiposity model. These findings are in line with another study that observed an increase in faecal SCFA concentration after six weeks of exercise training in lean but not in obese individuals.^51^ Moreover, a study in young adults (n = 39) has also reported a positive association between cardiorespiratory fitness and faecal butyric acid.^52^ Conversely, a negative association between step count and faecal SCFA has been reported in older individuals with insomnia (n = 49)^53^ and between accelerometer-assessed time spent in MVPA and faecal butyrate concentration in overweight and obese individuals (n = 124).^49^ The SCFA have been highlighted as one of the most beneficial gut microbiota products, with potential positive effects on gut barrier, insulin sensitivity, body weight, and systemic inflammation.^54^^,^^55^ Altogether, there are contrasting study results regarding the association between physical activity and SCFA metabolism, with some studies reporting BMI-dependent effects.

In the present study, SED was associated with higher abundance of *R. torques*, *R. gnavus*, *E. lenta*, and *Escherichia coli*. An elevated abundance of *R. gnavus* has been observed in obese individuals and the abundance decreased after a six-month program of weight loss.^56^ An increased abundance of the *Escherichia/Shigella* taxon was also reported in sedentary individuals in the Healthy Life in an Urban Setting cohort (HELIUS, N = 1334), the largest population-based study on physical activity and gut microbiota until the present study.^24^ A higher abundance of *E. coli* has been observed in individuals with atherosclerotic cardiovascular disease^57^ and users of the anti-diabetes medication metformin,^58^ suggesting a link between physical activity, gut microbiota, and cardiometabolic diseases. Due to different microbiota profiling methods, our findings cannot be directly compared with the HELIUS study. We confirmed though the positive associations between physical activity and *P. copri* and between SED and species in the genera *Erysipelatoclostridium* (e.g., *E*. *ramosum*) and *Lachnoclostridum* (e.g. *Lachnoclostridium* sp. HG3A.0655).

The median VPA in our study sample was only 0.06%. A previous study has suggested that as little as 15 min/week of VPA could reduce the risk for all-cause mortality by 18%^59^; therefore, effects of VPA could be expected even with little time spent in these activities. Vigorous-intensity activities include running and high-intensity sports, while moderate-intensity activities cover a wider range of activities, including brisk walking and bicycling at low speed, but also household chores.^60^ Future studies could further investigate the associations of subcategories of MPA with the gut microbiota.

With regards to the metabolic functional potential of the gut microbiota, we found that SED was associated with lower capacity for carbohydrate degradation, particularly from fibres, such as pectin and arabinoxylan. Likewise, a previous study has reported an increased abundance of carbohydrate degradation pathways in athletes.^61^ These findings could be due to lower fibre content in the diet of sedentary individuals, which we aimed to address by adjusting for fibre, whole grains, fruit and vegetables intake. A lower availability of fermentable carbohydrates in the distal gut can result in a reduction in saccharolytic bacteria and an increase in proteolytic bacteria.^35^ Intervention studies with standardized diet would be needed to disentangle physical activity associations from associations due to differences in dietary intakes.

For the modules in the microbiota-gut-brain axis, SED was negatively and MPA positively associated with the module for the heat-shock protein ClpB, that has been suggested to influence appetite regulation.^62^ SED was also associated with higher abundance of the GABA synthesis module and higher abundance of *E. coli*, one of the main GABA-producing bacteria in the gut.^63^ In line with those findings, we observed a negative association between MPA and *Bifidobacterium dentium*, another GABA-producing bacterium.^64^ Although lower plasma levels of GABA have been described in individuals with depression,^65^ recent studies found elevated levels in a mixed sample of medicated and non-medicated individuals with major depressive disorder.^66^^,^^67^

Several mechanisms are likely to be involved in the link between physical activity and the gut microbiota. Physical activity leads to shorter intestinal transit time.^17^ In the Estonian Microbiome Cohort (n = 3262), from 252 factors studied, gut emptying frequency explained the largest proportion of the interindividual gut microbiota variation.^39^ Additionally, physical activity has broad effects on the immune system, which could have repercussions for the gut microbiota. Exercise leads to an increase in mucosal immunoglobulin A,^68^ changes the composition of the gut-associated lymphoid tissue,^69^ and reduces the expression of inflammatory mediators in intestinal lymphocytes.^70^ Physical activity also reduces the splanchnic blood flow and increases the intestinal permeability.^18^ Furthermore, physical activity is associated with lower faecal concentration of bile acids,^19^ which can have substantial impacts on the gut microbiota composition.^71^

The strengths of this study are the accelerometer-based assessment of SED and physical activity phenotypes, the large sample of participants from the general population, and the high taxonomic resolution microbiome data. Moreover, we had access to comprehensive information on potential confounders. Some limitations apply. One concern is whether the associations described reflect the lifestyle of health-conscious individuals. Despite covariate adjustments, it is implausible to capture all dimensions of dietary intake and residual confounding may remain. In addition, information on diet and other potential confounders were self-reported and could cause reporting bias. Accelerometers measure absolute physical activity intensity, but relative intensity could be more clinically relevant. Standardized accelerometer cut-offs can misclassify low and high fitness individuals. Estimating relative intensity would though require data on individual maximal capacity, such as maximal oxygen uptake.^72^ Additionally, social desirability bias and adherence to the study instructions could affect accelerometer-based assessment. However, these misclassifications would be non-differential. The accelerometer needed to be removed during water-based activity and may also underestimate physical activity intensity during cycling, upper body activities, and weight-lifting.^28^ Our study was conducted in a Swedish population aged 50–65, thus generalizability to other populations is limited. Additionally, this study did not investigate the timing of physical activity throughout the day. It is suggested that gut microbiota may favour the practice of exercise by enhancing the enjoyment of physical activity^73^ or improving the host performance, which could be sources of reverse causation.^74^ The gut microbiota can also affect adiposity,^75^ which is negatively associated with physical activity. Lastly, the use of relative abundances entails the issues of compositional data, which can produce false–positive associations.^76^

In summary, sedentary behaviour and physical activity were associated with a large number of gut microbiota species and functional modules. Our findings can be used to guide research on the interplay between physical activity, the gut microbiota composition, and health outcomes. Studies investigating changes in the gut microbiota induced by physical activity with consequences for the host can contribute to characterize a healthy microbiota composition and pinpoint microbial candidates for intervention.

## Contributors

JGS, GBergström, LL, GE, JÄ, MO-M and TF obtained the funding for the study. GBaldanzi, SS-B, EEB, ÖE, UH, BK, and TF planned and designed the study. GBaldanzi carried out the statistical analyses with contribution from SS-B, KFD, and UH. GBaldanzi wrote the original draft of the manuscript with support from SS-B, BK, and TF. EEB and GBaldanzi accessed and verified the accelerometer data reported in the manuscript. SS-B and GBaldanzi accessed and verified the microbiome and the SCAPIS data reported. All authors contributed with the critical interpretation of the results and reviewing and editing the manuscript.

## Data sharing statement

The dataset supporting the conclusions of this article was provided by the SCAPIS Data access board and are not shared publicly due to confidentiality. Data will be shared upon reasonable request to the corresponding author only after permission by the Swedish Ethical Review Authority (https://etikprovningsmyndigheten.se) and by the SCAPIS Data access board (https://www.scapis.org/data-access/). The de-identified and de-hosted metagenomic sequences can be found in the European Nucleotide Archive under the accession code “PRJEB51353”. The statistical analyses R codes can be found at https://github.com/MolEpicUU/physicalactivity_gut.

## Declaration of interests

JÄ has served on the advisory boards for Astella, AstraZeneca and Boehringer Ingelheim and has received lecturing fees from AstraZeneca and Novartis, all unrelated to the present work. Remaining authors declare no competing interests.
