# Supplementary material for: Accelerometer-based physical activity is associated with the gut microbiota in 8416 individuals in SCAPIS
Source: eBioMedicine. 2024 Jan 31;100:104989. doi: 10.1016/j.ebiom.2024.104989 (PMC10844941; doi:10.1016/j.ebiom.2024.104989)
Supplement: Supplementary methods and Figs. S1–S3 [file mmc2.docx]

**ACCELEROMETER-based physical activity is associated with the gut microbiota in 8416 INDIVIDUALS in SCAPIS**

Gabriel Baldanzi^1^, Sergi Sayols-Baixeras^1,2^, Elin Ekblom-Bak^3^, Örjan Ekblom^3^, Koen F. Dekkers^1^, Ulf Hammar^1^, Diem Nguyen^1^, Shafqat Ahmad^1,4^, Ulrika Ericson^5^, Daniel Arvidsson^6^, Mats Börjesson^7,8^, Peter J. Johanson^9,10^, J Gustav Smith^11,12,13^, Göran Bergström^14,15^, Lars Lind^16^, Gunnar Engström^5^, Johan Ärnlöv^17,18^, Beatrice Kennedy^1^, Marju Orho-Melander^5^, Tove Fall^1^

# Supplemental Methods

## Faecal metagenomics

The gut microbiota analysis processing has been previously described.^1^ In summary, faecal samples were collected at home using the material provided at the first visit to the test centre. Participants were instructed to keep samples at the home freezer until the second visit to the study site, for further shipping to the central biobank for storage at -80°C. From the biobank, the sample boxes were shipped on dry ice in random order to Clinical Microbiomics A/S (Copenhagen, Denmark) for processing during 2019 and 2020.

DNA extraction was performed using NucleoSpin® 96 Soil kits (740787; Macherey-Nagel; Germany, batch number. 1903/001). Library preparation used the NEBNext® DNA Library Prep Kit (New England Biolabs). Sequencing was performed with Ilumina Novaseq 6000 (Illumina, USA) and generated an average read count of 25.3 million (SD = 3.8 million) read-pairs for Uppsala samples and 26.8 million (SD = 6.9 million) read-pairs for Malmö samples. Reads with adapter contamination, >10% ambiguous bases, >50% bases with Phred score <5, and reads mapped to human reference genome GRCh38 were removed. Reads were them mapped to metagenomic species as defined by Clinical Microbiomics A/S^2^ and species counts were normalized according to the read length. Taxonomic annotation was based on the NCBI RefSeq database (downloaded on 2 May 2021). For 12 samples, it was not possible to perform the faecal metagenomics (e.g. low DNA yield), these participants were excluded.

A downsized or rarefied species count matrix was also produced by random sampling, without replacement, from the subset of gene count table corresponding to the metagenomic species signature genes. The final read count for the downsized data was 210 430 read-pairs. One sample had significantly less reads assigned to the metagenomic species signature genes and was not included in the downsized matrix. The downsized matrix was used to calculate the alpha and beta diversity metrics. All other analyses were performed with the non-downsized data.

The functional potential of the gut microbiota was defined in terms of gut metabolic modules (GMM v1.07)^3^ and microbiota-gut-brain modules (MGB v1.0).^4^ To calculate the modules abundance, reads were mapped to Kyoto Encyclopedia of Genes and Genomes (KEGG) orthology (KO) database (<https://www.genome.jp/kegg/>) using EggNOG-mapper-software (v. 2.0.1).^5^ We removed one of the modules when there was an overlapped between GMM and MGB, thus we removed the modules MGB049, MGB 036, MF0030, MF0031, MF0032, MF0047, MF0075, MF0076, MF0082, MF0088, MF0089, MF0093, MF0094, MF0095. Next, we used Omixer-RPM v.0.3.2^6^ considering a minimum module coverage of 66.6%. We focused on species with a relative abundance >0·01% in ≥1% of individuals, and GMMs and MGBs present in ≥1% of individuals, resulting in 1335 species, 90 GMMs, and 44 MGBs for further analysis.

## Covariates

Covariate information was obtained from the SCAPIS questionnaire, and anthropometric measurements and fasting plasma samples collected during a study site visit. From the food frequency questionnaire MiniMeal-Q^7^, the mean daily intake of alcohol, vegetable and fruit, whole grain, protein (all g/day), and total energy (kcal/day) were estimated.^7,8^ Total daily fruit and vegetable intake was calculated based on questions about frequencies of fruit and vegetable intakes (including legumes) and portion sizes using weights per standard units^9^ and photo-options on vegetables presented to the participants. Added sugar intake was estimated by summing the intakes of sucrose and monosaccharides, followed by subtraction of the calculated mean intake of those sugars from common Swedish fruits and vegetables including juices (sucrose intake + fructose intake + glucose intake - fruit and berry intake×0.10 - vegetable intake×0.03 -juice intake×0.08).^10^ Protein was transformed to percentages of non-alcohol energy intake. Women with an estimated average energy intake ≤500 or ≥5000 kcal/day and men with an energy intake ≤550 or ≥6000 kcal/day were considered over or under-reporters of dietary intake and their dietary variables were set to missing. We categorized smoking status as current, former, or non-smoker, and highest achieved education level as incomplete compulsory education, complete compulsory education, secondary education, or university education. Country of birth was grouped as Scandinavia (Sweden, Denmark, Norway, or Finland), non-Scandinavian Europe, Asia, and other countries. For the variable “month when the accelerometer was worn” the categories June and July were merged because there were only two participants for July.

Data on dispensed prescribed medications were obtained from the Swedish Prescribed Drug Register held by the National Board of Health and Welfare, which includes information on all pharmacological agents prescribed in Sweden since 2005.^11^ We retrieved from the drug register information on medications prescribed within six months before the first study site visit for hypertension (Anatomical Therapeutic Chemical codes [ATC] C02, C03A, C03EA01, C08C, C09, and C07 – except C07AA07 sotalol)^12^, diabetes (ATC A10)^13^, dyslipidaemia (ATC C10), depression (ATC N06A), and anxiety (ATC N05B). If a participant reported in the questionnaire to have used a medication for hypertension, diabetes, or dyslipidaemia in the last two weeks, the participant was defined as a user for that medication even if a prescription had not been dispensed in the last six months. Because proton pump-inhibitors are sold without a prescription in Sweden, proton-pump inhibitor usage was defined as a measurable level of omeprazole or pantoprazole metabolites in the plasma.^1^ Antibiotic use was not present in the questionnaire data. Thus, antibiotic use was defined as a dispensed prescription (ATC code J01) up to three months before the first study site visit.

## References

1. Dekkers KF, Sayols-Baixeras S, Baldanzi G, Nowak C, Hammar U, Nguyen D, et al. An online atlas of human plasma metabolite signatures of gut microbiome composition. *Nat Commun* 2022; **13**: 5370.

2. Nielsen HB, Almeida M, Juncker AS, Rasmussen S, Li J, Sunagawa S, et al. Identification and assembly of genomes and genetic elements in complex metagenomic samples without using reference genomes. *Nat Biotechnol* 2014; **32**: 822–8.

3. Vieira-Silva S, Falony G, Darzi Y, Lima-Mendez G, Garcia Yunta R, Okuda S, et al. Species-function relationships shape ecological properties of the human gut microbiome. *Nat Microbiol* 2016; **1**: 16088.

4. Valles-Colomer M, Falony G, Darzi Y, Tigchelaar EF, Wang J, Tito RY, et al. The neuroactive potential of the human gut microbiota in quality of life and depression. *Nat Microbiol* 2019; **4**: 623–32.

5. Huerta-Cepas J, Forslund K, Coelho LP, Szklarczyk D, Jensen LJ, von Mering C, et al. Fast Genome-Wide Functional Annotation through Orthology Assignment by eggNOG-Mapper. *Mol Biol Evol* 2017; **34**: 2115–22.

6. Darzi Y, Falony G, Vieira-Silva S, Raes J. Towards biome-specific analysis of meta-omics data. *ISME J* 2016; **10**: 1025–8.

7. Christensen SE, Möller E, Bonn SE, Ploner A, Wright A, Sjölander A, et al. Two New Meal- and Web-Based Interactive Food Frequency Questionnaires: Validation of Energy and Macronutrient Intake. *J Med Internet Res* 2013; **15**: e109.

8. Christensen SE, Möller E, Bonn SE, Ploner A, Bälter O, Lissner L, et al. Relative Validity of Micronutrient and Fiber Intake Assessed With Two New Interactive Meal- and Web-Based Food Frequency Questionnaires. *J Med Internet Res* 2014; **16**: e59.

9. The Swedish National Food Administration SLV. *Vikttabeller* 1989; 1–49.

10. Ramne S, Alves Dias J, González-Padilla E, Olsson K, Lindahl B, Engström G, et al. Association between added sugar intake and mortality is nonlinear and dependent on sugar source in 2 Swedish population–based prospective cohorts. *Am J Clin Nutr* 2019; **109**: 411–23.

11. Wettermark B, Hammar N, Fored CM, Leimanis A, Otterblad Olausson P, Bergman U, et al. The new Swedish Prescribed Drug Register--opportunities for pharmacoepidemiological research and experience from the first six months. *Pharmacoepidemiol Drug Saf* 2007; **16**: 726–35.

12. Mubanga M, Byberg L, Egenvall A, Sundström J, Magnusson PKE, Ingelsson E, et al. Dog ownership and cardiovascular risk factors: a nationwide prospective register-based cohort study. *BMJ Open* 2019; **9**: e023447.

13. Delicano RA, Hammar U, Egenvall A, Westgarth C, Mubanga M, Byberg L, et al. The shared risk of diabetes between dog and cat owners and their pets: register based cohort study. *BMJ* 2020; **371**: m4337.

# Supplemental Figures

## Figure S1

Figure S1. **Spearman correlation between the accelerometer-based variables**. Additive log-ratio (alr) transformations were performed using low-intensity physical activity as the reference component (i.e., denominator). SED: percentage of time in sedentary behaviour; LIPA: percentage of time in low-intensity physical activity; MPA: percentage of time in moderate-intensity physical activity; VPA: percentage of time in vigorous-intensity physical activity; alr-SED: alr-transformed time in sedentary behaviour; alr-MPA: alr-transformed time in moderate-intensity physical activity; alr-VPA: alr-transformed time in vigorous-intensity physical activity.

## Figure S2

Figure S2. **Directed acyclic graph depicting the assumptions about the association between physical activity and the gut microbiota.**

## Figure S3

Figure S3. **Spearman** **correlation between the regression coefficients from the main model and from the adiposity model**. SED, MPA, and VPA were transformed using additive log-ratio (alr) transformation with time in low-intensity physical activity as the reference component (i.e., the denominator). The accelerometer variables were jointly analysed in the models. Main model: age, sex, alcohol intake, smoking, education, country of birth, study site, month of accelerometer wear, total energy intake and intake from proteins, fruit and vegetables, and whole grain, total accelerometer wear time, percentage of wear time on weekend, and faecal DNA extraction plate. Adiposity model: main model with additional adjustment for BMI and waist-hip ratio. SED: time in sedentary behaviour; MPA time in moderate-intensity physical activity; VPA: time in vigorous-intensity physical activity.
